# Genetic evidence linking the promoter region and 5’-UTR deletion of GNAS with pseudohypoparathyroidism type 1 C

**DOI:** 10.1186/s12920-026-02439-x

**Published:** 2026-07-28

**Authors:** Chung Lee, Yoon Ji Ahn, Sung-Chul Jung, Saeyoung Park, Ji Yeon Kim, Chang-Seok Ki, Woong-Yang Park, Sung Yoon Cho

**Affiliations:** 1 Department of Pathology, Yonsei University College of Medicine, Seoul, 03722 Korea; 2 Department of Pediatrics, Samsung Medical Center, Sungkyunkwan University School of Medicine, Seoul, 06351 Korea; 3 Department of Biochemistry, College of Medicine, Ewha Womans University, Seoul, 03760 Korea; 4 Department of Biomedical Science, CHA Stem Cell Institute, CHA University, Seongnam-si, 13496 Korea; 5 GC Genome, Yongin-si, 16924 Korea; 6 Samsung Genome Institute, Samsung Medical Center, Sungkyunkwan University School of Medicine, Seoul, 06351 Korea

**Keywords:** Pseudohypoparathyroidism 1 C, *GNAS*, Gs alpha, 5’ untranslated region

## Abstract

**Background:**

Pseudohypoparathyroidism (PHP) is a heterogeneous disorder caused by genetic or epigenetic changes at the *GNAS* locus on chromosome 20q13.3 leading to impaired cyclic adenosine monophosphate (cAMP)-dependent signaling pathway via the alpha-subunit of the stimulatory G protein (G_s_α). Unlike PHP type 1 A (PHP1A), which occurs due to *GNAS* variant and low G_s_α activity, the activity of G_s_α is normal in PHP type 1 C (PHP1C) despite the same phenotypic characteristics as PHP1A. However, the genetic cause of PHP1C is unknown.

**Methods:**

The likely causative variant of PHP1C was investigated using next-generation sequencing, and G_s_α protein levels and function were assessed in patient-derived cells by immunoblotting, immunocytochemistry and cAMP measurement.

**Results:**

We identified a *de novo* genomic deletion in a promoter region and 5’ untranslated region (UTR) of *GNAS (*5’ deletion of *GNAS)*, in a participant with PHP1C. Furthermore, although the mRNA and protein levels of *GNAS* were maintained, cAMP production was impaired in PHP1C, implicating a functional abnormality rather than quantitative changes.

**Conclusions:**

This novel 5’-UTR deletion of *GNAS* may underlie PHP1C and highlights the importance of assessing non-coding regions of GNAS in participants with PHP1C or PHP1A who lack pathogenic variants in conventional genetic tests.

**Supplementary Information:**

The online version contains supplementary material available at https://doi.org/10.1186/s12920-026-02439-x.

## Introduction

Pseudohypoparathyroidism (PHP) type 1 C (PHP1C) is clinically identical to PHP1A, sharing both the presence of Albright’s hereditary osteodystrophy (AHO) and multi-hormone resistance. However, unlike PHP1A, in vitro assessments have shown that the alpha-subunit of the stimulatory G protein (G_s_α) activity reveals no abnormality in patients with PHP1C [1]. Therefore, studies have postulated that, instead of a functional impairment of G_s_α, abnormalities in other components of the cyclic adenosine monophosphate (cAMP)-dependent signaling pathway, such as adenylyl cyclase (AC), inhibitory G proteins, or phosphodiesterases, might cause PHP1C [2]. However, the underlying genetic cause and the mechanism of PHP1C remains unknown.

Previously, four patients with PHP1C were reported in whom the disease was caused by *GNAS* variants affecting receptor coupling (p.L388R, p.E392X, p.E392K, p.Y391X in exon 13) [2, 3]. Further, six other patients with PHP1C were reported harboring epigenetic changes involving the *GNAS* locus [4, 5]. Similarly, epigenetic alterations of *GNAS* have been reported in four patients with hormone resistance and AHO-like features lacking *GNAS* coding mutations [2–4, 6]. However, most patients diagnosed with PHP1C showed neither epigenetic nor molecular genetic changes in *GNAS*, demonstrating that other pathogenic mechanisms might be responsible for PHP1C. We hypothesize that PHP1C is a single-gene disorder inherited in an autosomal dominant manner. Further, we speculated that the responsible gene product might be located functionally near G_s_α but before AC in the cAMP-dependent signaling pathway.

In this study, we investigated the functional significance of a novel deletion identified in the 5’ deletion of *GNAS* in a participant with PHP1C. Based on the results, we propose a diagnostic approach for patients with PHP1C or 1 A whose likely causative variant could not be identified using conventional genetic methods.

## Methods

### Subjects

Peripheral blood samples were drawn from the participant to assess multihormone resistance. X-ray imaging was performed to confirm the presence of AHO. Gene analysis and methylation-specific ligation-dependent probe amplification (MS-MLPA) of *GNAS* were performed using the peripheral blood samples. The activity of G_s_α from the erythrocyte membrane of the participant was analyzed using heparinized blood samples [7].

To investigate the likely causative variant of PHP, peripheral blood samples were drawn from the PHP1C Korean trio, the PHP1A Korean participant, the PHP1A Italian & Japanese participants, and a healthy Korean individual (wild-type). The variants were classified into five tiers according to the American College of Medical Genetics and Genomics/Association for Molecular Pathology (ACMG/AMP) guidelines [8].

### Reverse transcription PCR analysis

Total RNA was extracted from cells using the Qiagen RNeasy Mini Kit (Qiagen, Hilden, Germany). Complementary DNA (cDNA) was synthesized using Superscript II (Invitrogen, Carlsbad, CA, USA) and oligo(dT)20 primers at 42°C for 1 h, followed by incubation at 72°C for 15 min. Target cDNA sequences were amplified using premixed kits (Bioneer, Daejeon, Korea) under the following conditions: initial denaturation at 95°C for 5 min followed by 35 cycles of denaturation at 95°C for 30 s, annealing at 45–60°C for 45 s and extension at 72°C for 44 s. Normalized amounts of the products were separated on a 1.5% agarose gel and visualized by ethidium bromide staining. The forward and reverse primer sequences were as follows: *GNAS* primer-2: F, 5’- ATGGGCTGCCTCGGGA-3’ and R, 5’- CATGCTCATAGAATTCGGGAG-3’; *GNAS* primer-3 F, 5’- ATTGAAACCATTGTGGCC-3’ and R, 5’-CGTCAAACATGTGGAAGTTG-3’; and glyceraldehyde 3-phosphate dehydrogenase (GAPDH) F, 5’-TGGTAT CGTGGAAGGACTCA-3’ and R, 5’-CCTGCTTCACC ACCTTCTTG-3’.

### Participant cell line culture

To make stable cell lines derived from the participant, a participant with PHP1A and a healthy person, lymphocytes were infected with Epstein-Barr virus (EBV) through co-cultured with the EBV-positive Akata BL cell line. The EBV cell line was cultured in Minimum Essential Medium supplemented with 10% fetal bovine serum and 1% penicillin/streptomycin. The medium was replenished every 3 days. The cells were maintained in a 5% CO_2_ incubator at 37 °C and passaged at ratios of 1:2 to 1:6 using TrypLE (Invitrogen).

### cAMP measurement

To measure the increase cAMP levels, EBV-transformed lymphocytes from wildtype, PHP1A and PHP1C were treated forskolin (10µM) in 30 min, respectively. Intracellular cAMP levels were measured using a cAMP EIA system (GE Healthcare, Little Chalfont, UK) following the manufacturer’s instructions. All experiments were performed six times to ensure reproducibility.

### Western blotting

The cells were washed with phosphate-buffered saline (PBS), detached, and incubated with PRO-PREP buffer (iNtRON Biotechnology, Seongnam-si, Korea) containing a phosphatase inhibitor cocktail solution (#P3200, GenDEPOT, Barker, TX, USA) on ice. The lysates were collected by centrifugation at 13,000×g for 20 min at 4 °C, and equal amounts of protein from the supernatants were separated using sodium dodecyl sulfate-polyacrylamide gel electrophoresis (SDS-PAGE). After transferring the proteins onto polyvinylidene fluoride membranes (#P2938, Millipore, Burlington, MA, USA), the membranes were probed overnight at 4 °C with antibodies against G_s_α, G_s/olf_α (K-20 and A-5, Cat no. SC-823 and SC-55545, respectively; Santa Cruz, Inc., Dallas, Texas, USA) and GAPDH (1:5,000, Cat no. LF-PA0212; Ab-Frontier, Seoul, Korea) and then incubated with a goat anti-mouse IgG secondary antibody (cat. no. SC-2005; Santa Cruz Biotechnology, Inc., Dallas, Texas, USA). The blots were washed and developed using the enhanced chemiluminescence reagents SuperSignal West Dura (Cat no. 34076; Thermo Scientific, Waltham, MA, USA) according to the manufacturer’s instructions. The images were captured with a densitometric scanning instrument (LAS-3000, Fujifilm, Tokyo, Japan).

### Immunocytochemistry

The cells were fixed in 4% paraformaldehyde (15 min, room temperature) and washed thrice in ice-cold PBS. For permeabilization, the cells were incubated in PBS containing 0.25% Triton X-100 for 10 min and rinsed thrice in PBS. After blocking with 1% bovine serum albumin (Bovogen Biologicals, Keilor East, VIC, Australia) for 1 h, the cells were incubated overnight at 4 °C with an anti-GNAS antibody (K-20, Cat no. SC-823; Santa Cruz Biotechnology, Inc.). Following rinsing in PBS, Alexa Fluor 568-conjugated secondary goat anti-mouse antibodies (Gibco Life Technologies, Cat. No. A-11031) were applied for 1 h at room temperature in the dark. The cells were mounted and stained with Vectashield H-1200 containing DAPI (Cat no. H-1200; Vector Laboratories, Burlingame, CA, USA) and then observed with an Olympus BX51 phase-contrast microscope (Olympus, Tokyo, Japan).

### Next-generation sequencing

To evaluate the inherited gene variants causing PHP, exome libraries from trio genomic DNAs were enriched using an Agilent SureSelect Human All Exon Enrichment Kit V5 (Agilent Technologies, Santa Clara, CA, USA) and sequenced on an Illumina HiSeq2500 platform (Illumina Inc., San Diego, CA, USA). After the 5’ deletion of *GNAS* was curated, the genome and transcriptome were sequenced from the genomic DNA and RNA of the participant, respectively. The short reads were aligned to the human reference genome (hg19) using Burrow-Wheeler Aligner with the MEM algorithm (version 0.6.2) [9]. SAMtools (version 0.1.18) [10], Picard Tools (version 1.86, https://broadinstitute.github.io/picard/) and GATK (version2.4-7) [11], were used to analyze the aligned sequences. Single-nucleotide variants and InDels (insertions/deletions) were detected by the UnifiedGenotyper of GATK. RNA reads were aligned to the manipulated STAR reference using the STAR aligner (version 2.4.0) [12] in the two-pass mode. Additionally, the 5’ deletion of *GNAS* could be directly confirmed through Integrative Genomics Viewer (IGV) [13].

### Statistical analysis

The RT-PCR, Western blot and cAMP results were presented as mean ± SEM. Statistical significance of differences was determined using one-way analysis of variance followed by Mann-Whitney U test using PRISM version 10.4.0 (GraphPad Software Inc., San Diego, CA, USA). Statistical significance was set at *P* < 0.05.

## Results

### Diagnosis and the clinical information of the participant

The full-term Korean baby was born after an uneventful pregnancy as the only child of her parents. At 14 years of age, she was diagnosed with AHO and PHP when she presented at the Department of Pediatrics with a round face, short stature, brachymetacarpia of the 4th and 5th metacarpals, and obesity. She showed resistance to PTH, thyroid-stimulating hormone (TSH), and gonadotropin (secondary amenorrhea) (Supplementary Table 1). Her parents did not exhibit any AHO signs or hormone resistance. She had no abnormalities in either gene analysis or MS-MLPA of *GNAS.* We performed direct sequencing and MS-MLPA of *GNAS* twice and obtained the same results. To rigorously identify the likely causative variant, chromosome analysis (to exclude balanced translocation), chromosomal microarray (to exclude large deletion of *GNAS* and paternal disomy), and RT-PCR (to exclude deep intronic variants in *GNAS*) were performed, and all the results were normal. A paternity test was performed, and the results revealed the true family. The activity of G_s_α from erythrocytes was normal. Therefore, this participant was confirmed to have PHP1C. The clinical and biochemical features are described in detail in the supplementary Fig. 1.

### Identification of the 5’ deletion of *GNAS*

**Fig. 1:**
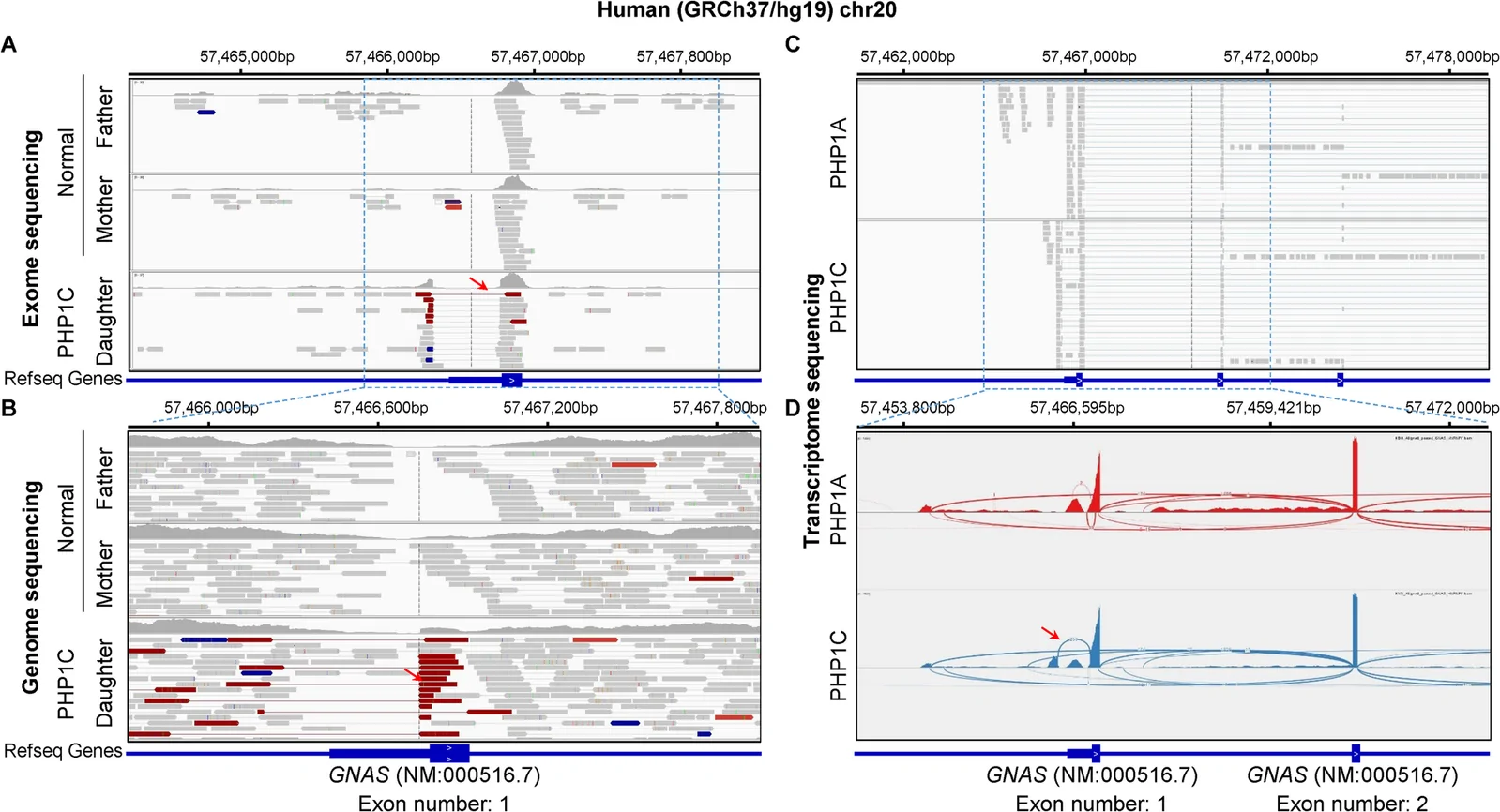
Integrative Genomics Viewer (IGV) was used to captured the 5’genomic deletion of *GNAS* which encoding G_s_α. from (**A**) Trio genome sequencing and (**B**) trio exome sequencing results with the option selected “View as pairs.” (**C**) Transcriptome sequencing results showing alternative splicing at exon 1 of *GNAS* in the participant with PHP1C. (**D**) A Sashimi plot at the *GNAS* promoter region. The red arrow shows split reads, expanded insert size, and alternative splicing suggesting genomic deletion or alternative splicing, respectively. The dotted red line and rectangle show the *GNAS* promoter region from different IGV zoom scales

We initially attempted to identify the causative gene variant using a conventional trio exome sequencing analysis but were unsuccessful. Subsequently, we thoroughly examined *GNAS* and observed discordant and split reads representing a 471-bp deletion (NC_000020.10(NM_000516.6):c.-471_-1del) in the 5’-UTR containing the CpG island where the promoter is located (Fig. 1A, Supplementary Fig. 2B). Because this region is GC-rich, it was insufficiently sequenced with few supporting reads. To overcome these challenges and accurately reconfirm the form of the variant, genome sequencing was performed on the participant, including both upstream and downstream regions. The results indicated a deletion in the 5’ deletion of *GNAS* and confirmed the absence of any additional structural *GNAS* variants (Fig. 1B).

### RNA and protein levels of *GNAS* and G_s_α activity

**Fig. 2:**
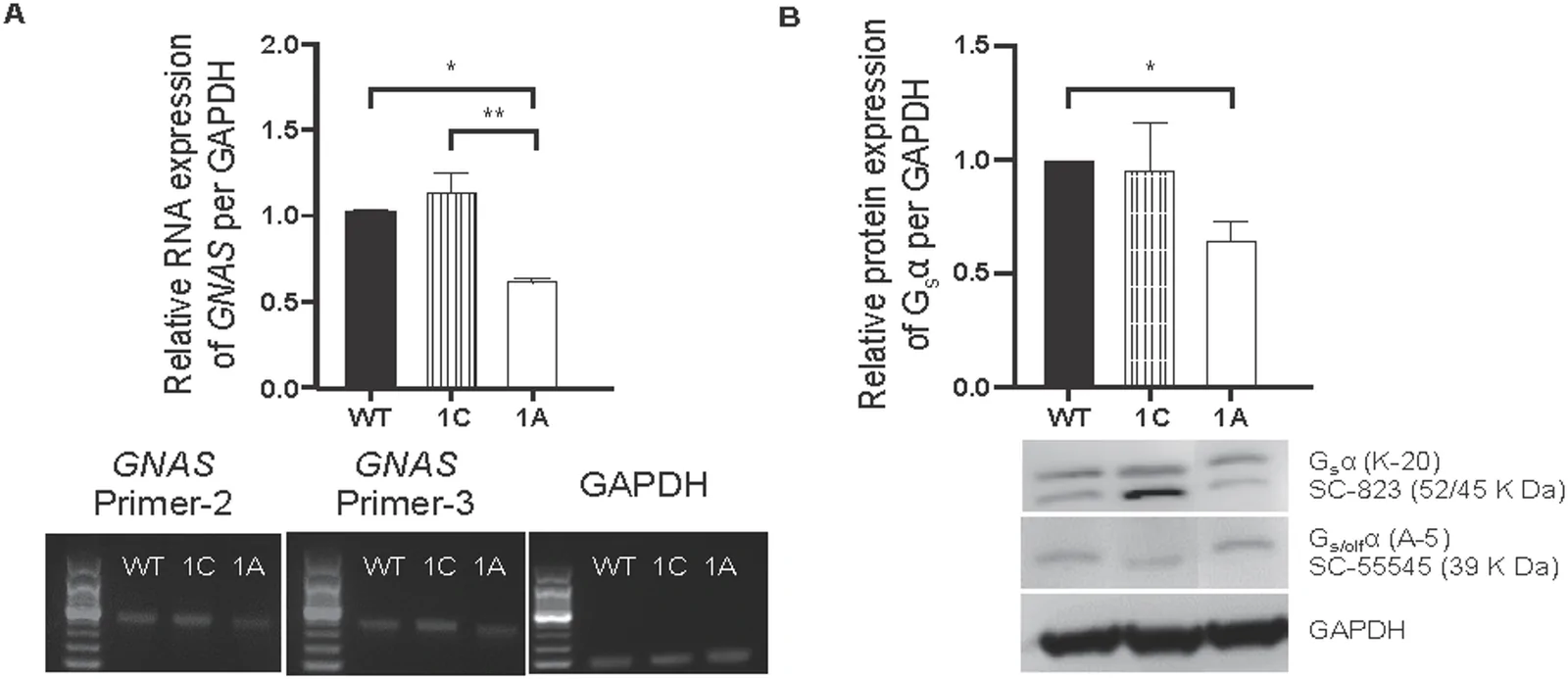
Quantification of the mRNA and protein levels of *GNAS*. (**A**) mRNA and (**B)** protein expression of G_s_α and G_s/olf_α in EB cell lines. Significance was determined using the Mann–Whitney U test. The data are presented as mean ± SEM. (*P < 0.05, **P < 0.01 compared to WT)

Transcriptome sequencing was performed to investigate how this genomic deletion affects the RNA levels. A sashimi plot of the 5’-UTR containing the deletion created using the IGV revealed an alternative splicing-like phenomenon upstream of the deletion compared with the PHP1A (Figs. 1 C and 1D). We hypothesized that this alternative splicing occurred in the mRNA generated from the allele with the deletion. However, RT-PCR analysis using two independent primers indicated no statistically significant differences in PHP1C compared with the wild type (Fig. 2A). In contrast, expression of *GNAS* in PHP1A was statistically significantly lower compared to PHP1C and the wild type.

Next, Western blotting was performed using two different antibodies to examine the differences at the protein level. Similar to the mRNA results, no statistically significant differences were observed in protein expression between PHP1C and the wild type while PHP1A showed a statistically significant decrease compared to the wild type as well known (Fig. 2B). Despite this, the results using the K-20 antibody indicated that the 45 kDa of G_s_α, also known as the short form, appeared to be overexpressed in PHP1C compared to the wild type. In summary, while alternative splicing was observed at the RNA level, no differences in mRNA or total protein levels were detected in PHP1C.

**Fig. 3:**
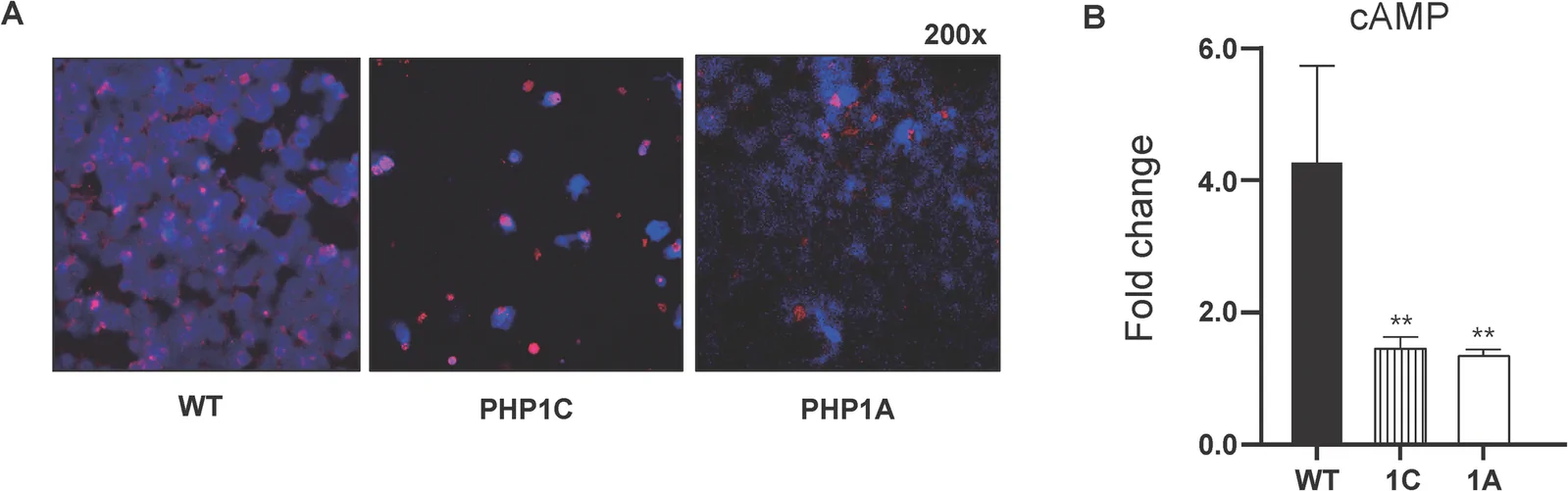
Incorrect G_s_α sub-localization and downregulated cAMP production in PHP1C. (**A**) Immunocytochemistry of G_s_α (K-20) with DAPI in the EB cell line. (**B**) Fold change of cyclic AMP increased in the EBV lymphocytes after forskolin treatment for 30 min. Significance was determined using the Mann–Whitney U test. The data are presented as mean ± SEM. (*P < 0.05, ** *P* < 0.01 compared to WT)

To investigate the potential effects of *GNAS* beyond quantitative differences, we performed immunocytochemistry to observe changes in the localization of G_s_α using a cell line derived from the lymphocytes of the participant. G_s_α (K-20) expression was predominantly observed at the cell membrane in the wild-type cells. However, in PHP1A and PHP1C, G_s_α was localized within the cytoplasm. G_s_α expression was decreased in PHP1A but maintained in PHP1C (Fig. 3A). However, cAMP levels were significantly reduced compared with the wild-type, suggesting that functional abnormalities might exist independent of quantitative changes (Fig. 3B). These findings indicate that while the 5’ deletion of *GNAS* does not affect the mRNA or protein levels in the participant, whereas it may prevent G_s_α from reaching the cell membrane, thereby impairing its ability to perform its primary function of binding G-protein coupled receptor (GPCR) and/or adenylate cyclase (AC) or generating cAMP (Fig. 3B).

## Discussion

PHP presents diagnostic challenges due to its clinical and genetic heterogeneity. This disorder encompasses various subtypes, each with distinct genetic variants and clinical manifestations, complicating accurate diagnosis [14–16]. Further, the complexity of *GNAS* and its imprinting effects require specialized testing and interpretation, making the diagnosis challenging [15]. PHP1C encompasses a group of diseases with the same clinical features as PHP1A but with normal G_s_α activity. However, it is hard to measure G_s_α activity in most institutions [1].

Despite the efforts to uncover the genetic basis of PHP1C, the underlying mechanism responsible for PHP1C remains unclear. Previously, four variants (p.L388R, p.E392X, p.E392K, p.Y391X) in exon 13 of *GNAS* were identified in six patients with PHP1C [2, 3], corresponding to iPPSD2 (inactivating PTH/PTHrP signaling disorder 2) within the classification proposed by the EuroPHP Network [5]. These variants affect the carboxyl-terminal residues of G_s_α, impairing receptor-mediated activation without affecting AC receptor-independent activation [2, 3]. In another study, six patients with PHP1C were found to have methylation defects instead of variants in the coding region of *GNAS* [4, 5]. Furthermore, *GNAS* methylation defects were reported in four patients with hormone resistance and AHO-like features lacking *GNAS* coding mutations [2–4, 6]. These findings correspond to iPPSD3 in the same classification, suggesting that epigenetic changes might contribute to the disease [4, 5]. However, most patients diagnosed with PHP1C showed neither epigenetic nor molecular genetic changes in *GNAS*, indicating that the pathogenic mechanisms underlying PHP1C might be diverse.

In this study, we suggest the 5’ deletion of *GNAS* as the likely causative variant of PHP1C. To the best of our knowledge, this is the first report identifying the genetic etiology of PHP1C, specifically related to an upstream deletion. Although the mRNA and protein levels of *GNAS* were maintained in PHP1C, unlike PHP1A, cAMP production was impaired in both diseases. Therefore, despite the adequate expression of proteins encoded by *GNAS*, the decreased cAMP levels might be due to the abnormal localization of G_s_α, disrupting its interactions with the G-protein coupled receptor (GPCR) or AC. Furthermore, a large amount of the short G_s_α isoform (45 kDa, G_s_α-S) was detected in PHP1C, which can compete with long G_s_α isoform (52 kDa, G_s_α-L) and decrease in AC activity [17], although this required further experimental validation. Notably, because this variant satisfies neither the iPPSD2 criterion (no loss-of-function mutation in *GNAS*) nor the iPPSD3 criterion (normal *GNAS* DMR methylation by MS-MLPA on two independent occasions), this participant would, under the current EuroPHP classification, be categorized as iPPSDx despite clear functional evidence of impaired G_s_α-mediated signaling. This suggests that iPPSDx may encompass a heterogeneous group of patients harboring as-yet-uncharacterized regulatory *GNAS* defects, and we propose that this deletion represents a candidate basis for a distinct, non-coding regulatory subtype within this framework.

Promoter abnormalities have been implicated in several genetic disorders. For instance, in Lynch syndrome, promoter methylation or deletion in *MLH1* results in the silencing of this critical mismatch repair gene, predisposing individuals to colorectal and other cancers [18, 19]. Similarly, in Prader-Willi syndrome (PWS), a microdeletion in the imprinting center (encompassing the *SNRPN* promoter) disrupts the paternal imprinting process. This leads to the loss of expression of multiple genes in the 15q11-q13 region [20], contributing to the characteristic neurodevelopmental and metabolic features of the syndrome. In contrast, Peery et al. revealed that the promoter deletion of *SNRPN* does not cause the imprinting defect [21]. Similarly, the promoter deletion identified in our participant did not affect the RNA or protein levels of *GNAS*. This difference may be attributed to the complex transcriptional landscape of *GNAS*, which generates multiple transcripts. Therefore, instead of relying on the original transcription-binding region of the deleted promoter, the gene might have utilized alternative promoters, such as the upstream promoter predicted by the Eukaryotic Promoter Database [22] (Supplementary Fig. 2B) or the *GNAS* A/B promoter, to maintain transcription. This compensatory mechanism, however, was not directly tested in this study; functional promoter assays, such as luciferase reporter assays comparing wild-type and deleted *GNAS* promoter constructs, represent an important direction for further study to experimentally validate this hypothesis.

The biological significance of the alternative splicing-like event detected upstream of the deletion also merits consideration. Because this event maps to the 5’-UTR of *GNAS* exon 1, upstream of the translational start codon (the deletion itself, NC_000020.10(NM_000516.6):c.-471_-1del, lies entirely within the 5’-UTR), it is unlikely to alter the downstream open reading frame or generate a novel protein isoform. We therefore favor the interpretation that this represents a cryptic splice site or altered transcription-start-site usage confined to the non-coding region, which could influence transcript stability or translational efficiency rather than generating an aberrant protein product. Notably, this interpretation does not conflict with our RT-PCR results: both primer sets used to quantify *GNAS* transcript levels (*GNAS* primer-2 and primer-3) anneal at or downstream of the translation start codon and were therefore not designed to detect a splicing difference confined to the 5’-UTR. The unchanged total *GNAS* mRNA and protein levels observed by RT-PCR and western blotting (Fig. 2) thus reflect normal coding-region transcript abundance and translation, which is fully compatible with an altered 5’-UTR splicing pattern further upstream. Nonetheless, we cannot formally exclude a functional consequence of this alternative transcript, and techniques such as 5’ RACE, 5’-UTR-specific RT-PCR, or long-read transcript sequencing spanning the deleted region would be needed to directly characterize its structure and translational fate in future studies.

The differential expression and potential functional divergence of the G_s_α isoforms emphasize the complexity of GNAS-mediated signaling in various tissues. While both the G_s_α-S and G_s_α-L can activate AC and regulate cAMP levels, studies have suggested that G_s_α-S can potentially compete with G_s_α-L, reducing the efficiency of guanosine diphosphate (GDP) release and diminishing the receptor coupling activity [23]. The distinct expression patterns of the G_s_α isoforms indicate their tissue-specific roles in cellular signaling. This raises the intriguing possibility that the structural differences, such as the 15-amino acid N-terminal insert in G_s_α-L, may influence receptor coupling or interactions with other effectors beyond the classical cAMP signaling pathways. Furthermore, the observed shifts in the G_s_α isoform ratios during development, aging, or metabolic stress implicate their involvement in adaptive cellular responses. Understanding these isoform-specific functions could elucidate the pathophysiology of *GNAS*-related diseases and facilitate more targeted therapeutic approaches, particularly in conditions where isoform dysregulation contributes to disease pathogenesis [17]. The possible imbalance between the Gsα-L and Gsα-S isoforms was inferred from total-protein immunoblotting alone (Fig. 2B), but isoform-specific transcript analyses (e.g., isoform-specific RT-PCR distinguishing Gsα-L and Gsα-S transcripts) were not performed in this study and will be required to confirm this hypothesis.

The abnormal G_s_α localization observed in K-20 immunohistochemistry appears to play a crucial role in the pathogenesis of PHP1C. The translocation of G_s_α between the membrane and the cytoplasm is influenced by cellular signaling events. For instance, studies have shown that certain treatments can cause G_s_α to move from lipid rafts to non-raft regions of the membrane, altering its signaling capabilities [24]. However, specific data for differentiating the subcellular localization dynamics between G_s_α-S and G_s_α-L remain limited. Both G_s_α-S and G_s_α-L are primarily localized at the plasma membrane, engaging in GPCR-mediated signaling. Impaired membrane localization can disrupt cAMP signaling, contributing to conditions like PHP. Two possible hypotheses can be proposed. The First is ‘the G_s_ protein complex imbalance hypothesis’. The protein product of the *GNAS* gene, G_s_α, forms a G_s_ protein complex by binding to β and γ subunits. The stoichiometric ratio among these subunits may be critical. If G_s_α levels are too low, it may impair proper Gs complex formation. Conversely, if G_s_α levels are too high, it could lead to the formation of Gs complexes containing two or three G_s_α subunits, which might fail to localize to the cell membrane and instead accumulate in the cytoplasm. The second is ‘the abnormal G_s_α formation hypothesis’. Along with the normal α subunit, an abnormal-sized G_s_α subunit might also be produced, leading to the formation of abnormal G_s_ complexes [25]. This hypothesis is supported by Fig. 3, which shows that in K-20 immunohistochemistry, red fluorescence intensity is significantly higher in PHP1C compared to the wild type. Since the *GNAS* gene is an imprinted gene, and its gene regulation is highly complex, further studies are required to validate these hypotheses.

In conjunction with this study, additional exome sequencing was performed on 8 Italian and 3 Japanese participants with PHP1A who did not possess *GNAS* variants by Sanger sequencing. Upon reexamining *GNAS*, we discovered that certain regions intended for analysis were not covered due to the limitations of the NGS technique, specifically the GC-rich regions. Two heterozygous variants in exon 1 of *GNAS* were identified and interpreted as pathogenic following ACMG guideline in two participants respectively (NM_000516.6:c.1 A > C (NP_000507.1:p.?) and NM_000516.6:c.91 C > T (NP_000507.1:p.Gln31Ter)), indicating that exon 1 is not well-covered by conventional sequencing methods. Please refer to the supplementary data for detailed information regarding the multinational study.

The main problem that this study intended to solve was how to sequence exon 1 of *GNAS* and its GC-rich promoter region in participants suspected of PHP1A/1 C. Sequencing was successful in the participant, possibly because this deletion lowered the GC content. As for genetically undefined PHP1C (no detected abnormalities in direct sequencing and MS-MLPA of *GNAS*), the upstream region and exon 1 of the *GNAS* gene should be checked carefully. Our hypothesis is that participants with PHP1C may have a heterozygous deletion in a similar region (5’ deletion of *GNAS*) as seen in the participant (NC_000020.10(NM_000516.6):c.-471_-1del), and the size and margin of the deletion can be different for each person.

This study is the first to identify the likely causative variant related to an upstream deletion for PHP1C, indicating the importance of examining the non-coding regions of *GNAS*. It would be interesting to determine the prevalence of this deletion in other populations and to establish whether it contributes to susceptibility to PHP. As a limitation, this study is based on data from a single participant with PHP1, so future research is needed to validate these findings and explore the functional implications of this genetic alteration.

## Supplementary Information

Supplementary Material 1. Supplementary Table 1

Supplementary Material 2. Supplementary Fig. 1

Supplementary Material 3. Supplementary Fig. 2

## Data Availability

No datasets were generated or analysed during the current study.
